# Exosomal communication: a pivotal regulator of bone homeostasis and a potential therapeutic target

**DOI:** 10.3389/fphar.2024.1516125

**Published:** 2024-12-23

**Authors:** Qian-Yun Ye, Yan Cui, Hao-Yu Wang, Ling-Yu Li, Jian-Bing Chen, Xiao-Feng Zhu, Zhi-Jian Xue, Rong-Hua Zhang

**Affiliations:** ^1^ Department of Traditional Chinese Medicine, First Affiliated Hospital of Jinan University, Guangzhou, China; ^2^ College of Traditional Chinese Medicine, Jinan University, Guangzhou, China; ^3^ College of Pharmacy, Jinan University, Guangzhou, China; ^4^ Guangdong Provincial Key Laboratory of Traditional Chinese Medicine Informatization, Jinan University, Guangzhou, China; ^5^ Cancer Research Institution, Jinan University, Guangzhou, China

**Keywords:** exosome, extracellular vesicle, bone homeostasis, osteoporosis, osteoarthritis

## Abstract

Bone homeostasis encompasses two interrelated aspects: bone remodeling and cartilage metabolism. Disruption of bone homeostasis can lead to the development of metabolic bone diseases such as osteoporosis and osteoarthritis. The maintenance of bone homeostasis is a complex process that does not solely rely on the functions of the bone tissue itself. In fact, bone tissue is not an isolated entity; it is closely connected to other tissues in the body via exosomes. Within this interconnectivity, exosomes derived from both bone and non-bone cells interfere with each other, forming a complex regulatory network. Therefore, with cell origin as the guiding principle, we have delineated the bone regulatory network of exosomes, elaborated on the specific roles and regulatory mechanisms of exosomes derived from common cell types (cells within the skeletal microenvironment, stem cells from extra-osseous tissues, vascular-derived cells, muscle-derived cells, and neurogenic cells) in bone formation, bone resorption, and cartilage metabolism. We have also discussed the challenges faced in the field of exosome research related to bone homeostasis, unveiled the critical role of exosomes in maintaining bone homeostasis, and proposed that exosomes could serve as highly valuable therapeutic targets for metabolic bone diseases.

## 1 Introduction

Bone homeostasis refers to the state of maintaining the overall structure and functional stability of the skeletal system. It encompasses two interrelated aspects: bone remodeling and cartilage metabolism. Bone remodeling involves the continuous update and repair of bone morphology and structure, including the dynamic balance between bone formation and bone resorption, while cartilage metabolism pertains to the processes of cartilage synthesis, degradation, and the maintenance of normal joint cartilage function (Salhotra et al., 2020; Boyde, 2021; Cardoneanu et al., 2022). The maintenance of bone homeostasis relies on the mediation of various cell types within the skeletal microenvironment, such as bone marrow mesenchymal stem cells, osteoblasts, osteoclasts, osteocytes, and chondrocytes (Kim et al., 2020; Vig and Fernandas, 2022), while also being regulated by cells outside the skeletal microenvironment (Florencio-Silva et al., 2015). This balance may be lost with aging or due to certain pathological factors, leading to the development of bone metabolic diseases such as osteoporosis (OP) and osteoarthritis (OA) (Feng and McDonald, 2011).

Exosomes are nanoscale membrane-bound lipid vesicles secreted by cells, containing bioactive substances such as proteins, RNAs, lipids, and cytokine receptors. They play a significant role in intercellular, interorgan, and systemic communication through paracrine or endocrine signaling pathways (Tkach and Thery, 2016; Wang et al., 2022). Exosomes can circulate in the body and may target cells via receptor-ligand interactions. Additionally, they can be internalized by target cells through endocytosis, delivering their cargo and activating related signaling pathways (Qin and Dallas, 2019; Mathivanan et al., 2010). Bone-derived and non-bone-derived cells can communicate with each other via exosomes, targeting the source cells, adjacent cells, or reaching distant organs through the circulation. This promotes multiple cascades of intracellular or intercellular signaling pathways, thereby regulating the skeletal microenvironment (Zhang L. et al., 2023).

In recent years, research on the role of exosomes in regulating bone homeostasis has been increasing, but the understanding of their regulatory network remains unclear. In this review, we have used cell origin as a unit, ranging from cells within the skeletal microenvironment to stem cells from non-bone tissues, vascular-derived cells, muscle-derived cells, and neurogenic cells. We have elucidated the intricate interactions among different cell populations and explored the effects of exosomes in mediating bone remodeling, cartilage regeneration, inflammatory responses, and immune regulation in bone homeostasis. By organizing and refining the regulatory network of exosomes from common cell sources on bone homeostasis, this review provides a theoretical basis for the study of bone metabolic diseases and holds clinical significance for their diagnosis and treatment.

It is worth noting that the concepts of “exosomes” and “extracellular vesicles (EVs)” are continuously being updated by organizations such as the International Society for Extracellular Vesicles (ISEV), and there is an ongoing effort to standardize the terminology. This process may involve differing viewpoints and methodologies, leading to variations in the use of terms. To present a more comprehensive view of the research landscape, we have also included an analysis of high-quality studies with the keyword “Extracellular vesicle”.

## 2 Exosome derived from cells in bone microenvironment

**FIGURE 1:**
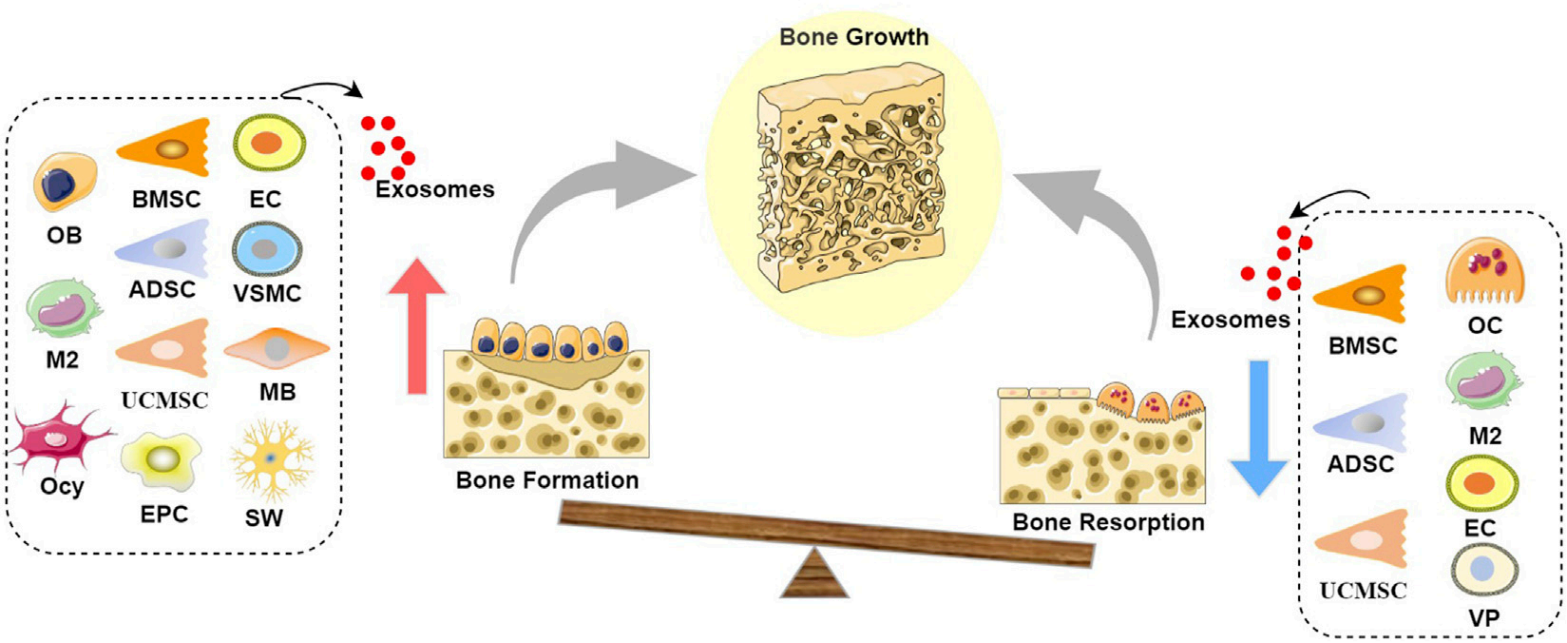
Schematic diagram of the role of exosomes in bone growth: Exosomes derived from BMSCs, UCMSCs, ADSCs, EPCs, OBs, M2s, Ocy, ECs, VSMCs, and MBs can promote bone formation, while exosomes derived from BMSCs, UCMSCs, ADSCs, OCs, M2s, ECs, and VPs can inhibit bone resorption, leading to a state where bone formation exceeds bone resorption, thereby resulting in positive bone growth. It is noteworthy that both OB-Exos and OC-Exos have negative feedback mechanisms; at certain stages, mature OB-Exos can inhibit osteoblast differentiation to prevent excessive bone formation, while mature OC-Exos can conversely promote bone formation, thereby achieving the dynamic transition between bone formation and bone resorption phases in the bone remodeling process. (Abbreviation: BMSC, Bone marrow mesenchymal stem cell; UCMSC, umbilical cord mesenchymal stem cell; ADSC, adipose-derived stem cell; EPC, endothelial progenitor cell; OB, Osteoblast; M2, M2 macrophages; Ocy, Osteocyte; EC, vascular endothelial cell; VSMC, vascular smooth muscle cell; MB, myoblasts; OC, Osteoclast; VP, vascular pericytes).

**FIGURE 2:**
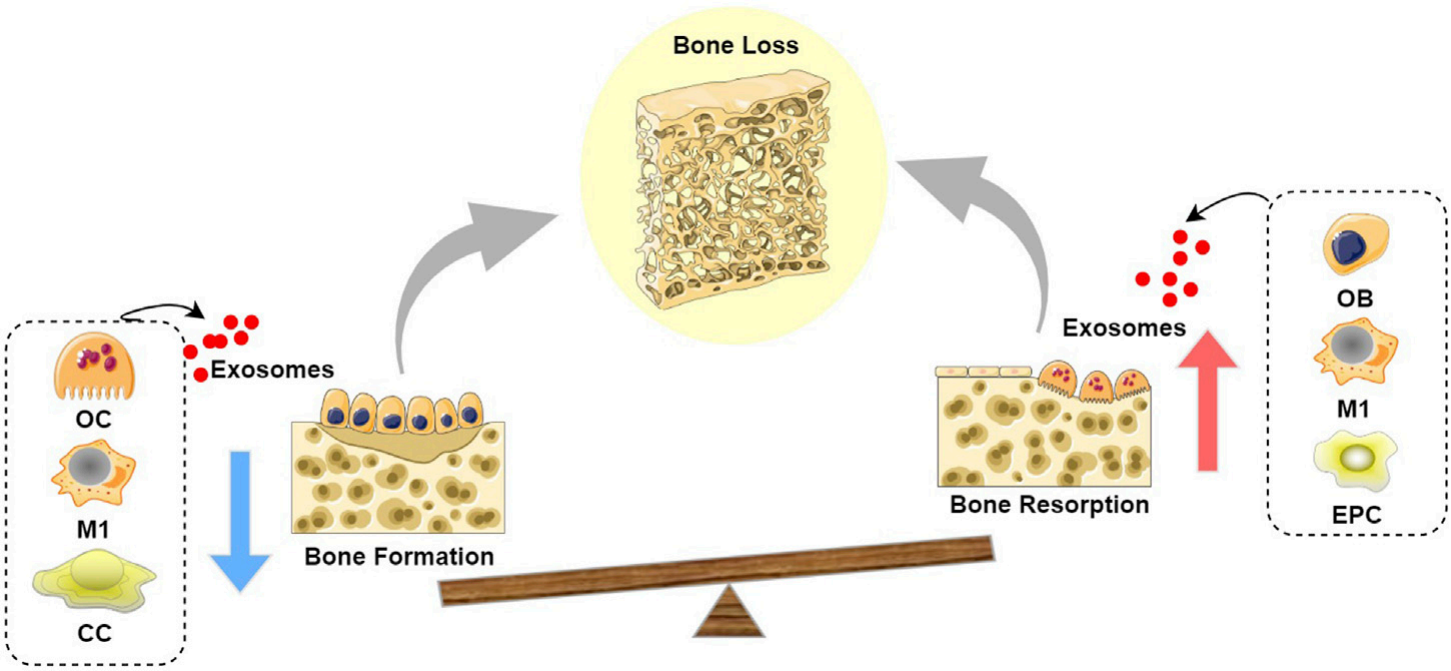
Schematic diagram of the role of exosomes in bone loss: Exosomes derived from OC, M1, and CC inhibit bone formation, while exosomes derived from OB, M1, and EPC promote bone resorption, leading to a state where bone resorption exceeds bone formation, thereby resulting in bone loss. (Abbreviation: OC, Osteoclast; M1, M1 macrophages; CC, chondrocyte; OB, Osteoblast; EPC, endothelial progenitor cell).

**FIGURE 3:**
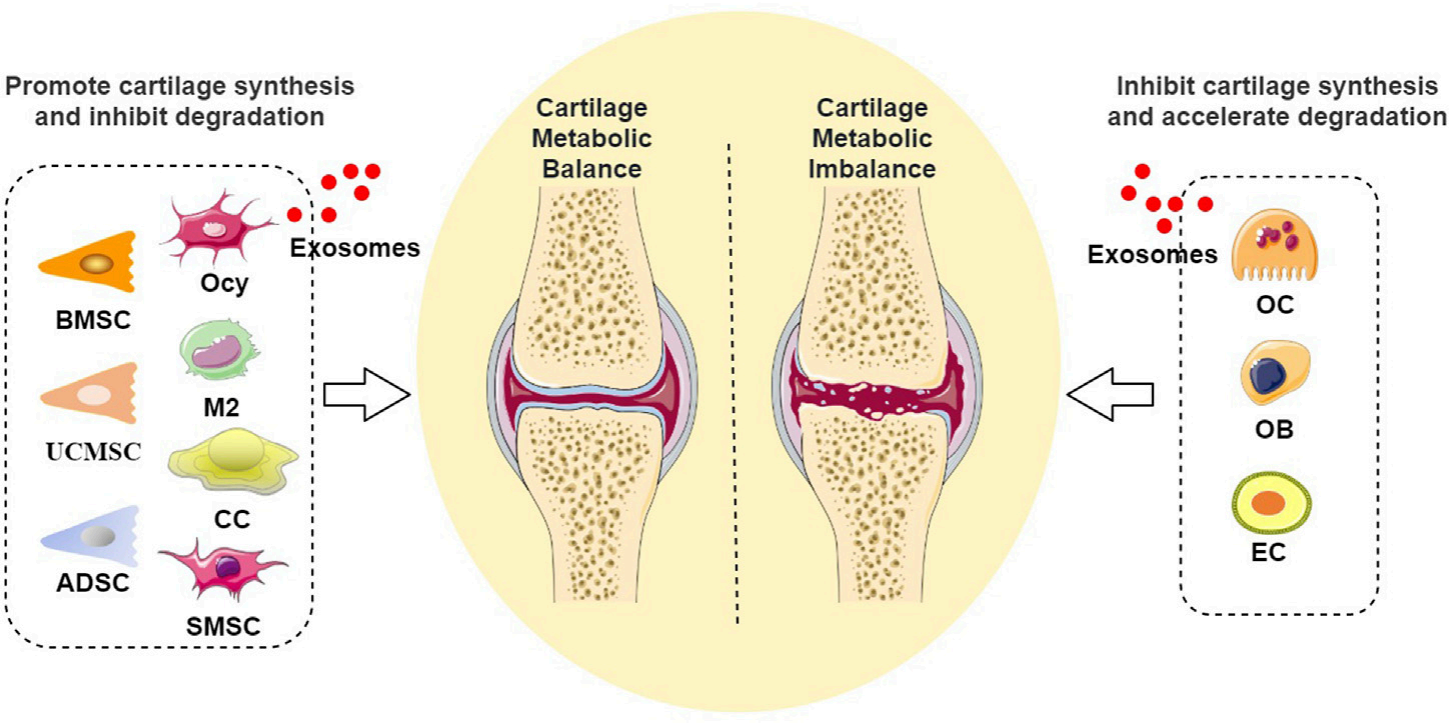
Diagram of the role of exosomes in cartilage metabolism: Exosomes derived from BMSC, UCMSC, ADSC, Ocy, M2, CC, and SMSC promote cartilage synthesis and inhibit degradation, while exosomes derived from OC, OB, and EC inhibit cartilage synthesis and accelerate degradation. (Abbreviation: BMSC, Bone marrow mesenchymal stem cell; UCMSC, umbilical cord mesenchymal stem cell; ADSC, adipose-derived stem cell; Ocy, Osteocyte; M2, M2 macrophages; CC, chondrocyte; SMSC, synovial mesenchymal stem cell; OC, Osteoclast; OB, Osteoblast; EC, vascular endothelial cell).

Bone marrow mesenchymal stem cells, osteoclasts, osteoblasts, osteocytes, chondrocytes, and macrophages are resident cells within the skeletal microenvironment. They maintain the dynamic balance of bone and cartilage metabolism through a sophisticated intercellular communication network. In this section, we provide a detailed description of the role of exosomes derived from cells within the skeletal microenvironment in bone homeostasis (illustrated in Figures 1–3). These exosomes carry a variety of bioactive molecules and play a crucial role in regulating cell behavior and responding to physiological or pathological signals.

### 2.1 Bone marrow mesenchymal stem cell-derived exosomes

Bone marrow mesenchymal stem cell-derived exosomes (BMSC-Exos) regulate bone formation by directly affecting the activity of the osteoblastic lineage, with their mechanisms of action involving multiple signaling pathways. Studies have shown that BMSC-Exos can activate osteogenic differentiation through the BMP-2/SMAD1/RUNX2 signaling pathway, enhancing the osteogenic potential of mouse embryo osteoblast precursor cells MC3T3-E1 *in vitro*, and promoting the expression of osteogenesis-related genes such as BMP2, SMAD1/5, RUNX2, OGN, OPN, and OCN in target bone tissue *in vivo* (Zhang et al., 2020). BMSC-Exos also facilitate the growth cycle of the osteoblastic line hFOB 1.19 by activating the JNK/MAPK signaling pathway, accelerating cell proliferation (Zhao et al., 2018). BMSC-Exos also exhibit a promotional effect on the osteogenic differentiation of their source cells. BMSC-Exos upregulate the expression of osteogenic-related genes such as TGF-β1, BMP9, Runx2, OSX, and OCN (Tsao et al., 2017; Narayanan et al., 2016). MicroRNAs (miRNAs) are key regulatory factors in the influence of BMSC-Exos on bone formation. It has been reported that miR-668-3p, miR-27a, miR-196, miR-206, miR-335, miR-150-3p, and miR-136-5p are enriched in BMSC-derived exosomes and play a role in promoting osteoblast proliferation and differentiation (Qiu et al., 2024; Qin et al., 2016; Qiu et al., 2021; Hu et al., 2021; Yu H. et al., 2021). BMSC-Exos can also upregulate osteogenic miRNAs (Hsa-miR-146a-5p, Hsa-miR-503-5p, Hsa-miR-483-3p, and Hsa-miR-129-5p) or downregulate anti-osteogenic miRNAs (Hsa-miR-32-5p, Hsa-miR-133a-3p, and Hsa-miR-204-5p) through the activation of the PI3K/AKT and MAPK signaling pathways (Zhai et al., 2020). Other non-coding RNAs contained within BMSC-Exos have also been confirmed to possess osteoinductive capabilities. The MALAT1 lncRNA contained in BMSC-Exos induces osteoblast differentiation by sponging miR-34c and activating SATB2, which in turn activates the transcription factors RUNX2 and ATF4 (Yang et al., 2019). Additionally, exosomal circHIPK3 promotes mitochondrial autophagy in MC3T3-E1 cells through the miR-29a-5p/PINK1 axis, thereby playing a crucial role in osteogenic differentiation (Ma et al., 2023).

BMSC-Exos exert an anti-bone resorption effect by inhibiting the migration of osteoclast precursors, suppressing osteoclast formation and excessive activation, and improving bone metabolism in OP mice. This mechanism may be related to the delivery of USP7 and the promotion of USP7-mediated deubiquitination and stabilization of YAP1 protein (Yin et al., 2023; Wang X. et al., 2023). BMSC-Exos produced under the stimulation of bioactive glass nanoparticles further enhance their ability to inhibit osteoclast differentiation (Yang et al., 2022). Non-coding RNAs in BMSC-Exos play a significant role in osteoclast differentiation. MiR-31a-5p targets RhoA, which jointly inhibit osteoclast differentiation and reduce bone resorption (Xu R. et al., 2018). However, aged BMSCs suffer from impaired mitochondrial metabolism and pluripotency, and they release a high amount of lncRNA-NEAT1, which can regulate miR-27b-3p through paracrine transfer, promoting osteoclast differentiation, and enhancing bone resorption activity (Zhang et al., 2022).

BMSC-Exos can reduce cartilage degradation through anti-inflammatory effects while increasing matrix synthesis by promoting chondrocyte proliferation (Ni et al., 2020). Studies have shown that BMSC-Exos can ameliorate the increase of MMP13 induced by inflammatory cytokines in chondrocytes, eliminate damage to mitochondrial membrane potential, and reduce chondrocyte degeneration and apoptosis (El-Din et al., 2024; Arévalo-Turrubiarte et al., 2022; Qi et al., 2019; Cosenza et al., 2017). Moreover, in an OA mouse model, BMSC-Exos have been demonstrated to reduce articular cartilage damage and subchondral bone degeneration, promoting cartilage regeneration at the histological level (Fazaeli et al., 2021; Cosenza et al., 2017; Chen P. et al., 2019). Exosomal miRNAs derived from BMSCs are involved in the regulation of OA pathological processes. The highly expressed miR-26a-5p in BMSC-Exos has been proven to interact with PTGS2, reducing OA inflammatory damage (Jin et al., 2020). TGF-β1 regulates SP1 through miR-135b in BMSC-Exos to promote chondrocyte proliferation, thereby facilitating cartilage repair (Wang et al., 2018). BMSC-derived exosomal miR-92a-3p can promote chondrocyte proliferation and matrix synthesis through the Wnt pathway (Mao et al., 2018). BMSC-derived exosomal SNHG7 inhibit IL-1β-induced inflammation and ferroptosis in chondrocytes through the miR-485-5p/FSP1 axis (Wang Y. et al., 2024). BMSC-Exos also promote the polarization of M1 macrophages to M2 by delivering lncRNA TUC339, enhancing chondrocyte activity (Shen et al., 2023). Additionally, exosomal miR-127-3p, and miR-206 derived from BMSCs also mediate the anti-inflammatory response and improve chondrocyte metabolism (Dong et al., 2021; Huang et al., 2021).

### 2.2 Osteoclast-derived exosomes

Osteoclast-derived exosomes (OC-Exos) are involved in the negative regulation of bone formation. The level of serum exosomal miR-214 is elevated in OP patients and ovariectomy-induced mice, and further studies have indicated that this is associated with the paracrine function of osteoclasts. Osteoclasts recognize osteoblasts through EphrinA2 and EphA2 and release miR-214-3p into osteoblasts, which can target ATF4, inhibiting osteoblast differentiation and bone formation. This trend can be reversed by inhibiting exosome release using delivery of Rab27a siRNA or by inhibiting miR-214-3p with antagomir (Sun et al., 2016; Deng et al., 2015; Wang et al., 2013). Another noteworthy miRNA in OC-Exos is miR-23a-5p. It has been reported that miR-23a-5p is highly expressed in RANKL-induced RAW 264.7 cell exosomes and can significantly inhibit osteoblast activity by suppressing Runx2 and promoting YAP1-mediated MT1DP (Yang J. X. et al., 2020). Additionally, proteins enriched in OC-Exos, such as SEMA4D, are upregulated during osteoclast differentiation, and their binding to the receptor Plexin-B1 on osteoblasts leads to the activation of the small GTPase RhoA, which inhibits bone formation by suppressing IGF-1 signaling and regulating osteoblast motility (Negishi-Koga et al., 2011). OC-Exos enriched with lncRNA AW011738 inhibit osteoblast differentiation through the miR-24-2-5p/TREM1 axis (Liu J. et al., 2024). Indeed, it is intriguing to note that OC-Exos appear to exert a complex dual effect in the regulation of osteoblast activity. The study by Ikebuchi and colleagues shows that RANK carried by mature osteoclast-derived exosomes (RAW 264.7 cells stimulated with GST-sRANKL for 60–120 h) can bind to RANKL on the surface of osteoblasts. This interaction triggers reverse signaling of RANKL, which in turn activates the key transcription factor RUNX2, thereby promoting bone formation (Ikebuchi et al., 2018). Furthermore, mature osteoclast-derived exosomes (Bone marrow macrophages stimulated with RANKL for 60–120 h) can be engulfed by MC3T3-E1 cells, and facilitate the mineralization of these cells through RANKL-mediated reverse signaling (Ma et al., 2019). This process underscores the intricate communication between osteoclasts and osteoblasts, where exosomes act as vehicles for signaling molecules that can either inhibit or promote osteoblast activity, depending on the context and stage of osteoclast maturation.

Osteoclasts act in a paracrine manner to stimulate osteoblasts or other non-osteoclast cells to produce factors that regulate osteoclast differentiation, participating in bone remodeling. Exosomes from osteoclast precursors (Bone marrow macrophages induced by CSF-1) can stimulate an increase in the number of osteoclasts in mouse bone marrow, whereas an equivalent number of exosomes from mature osteoclasts (Bone marrow macrophages induced by CSF-1 and sRANKL) inhibit osteoclast formation, which may be related to the enrichment of RANK in OC-EVs rather than in pre-OC-EVs (Yuan et al., 2018; Huynh et al., 2016; Holliday et al., 2017; Huynh et al., 2016). A study analyzing the miRNA expression profile of OC-EVs found reduced levels of miR-22-3p, miR-26a-5p, miR-27a-3p, miR-29a-3p, and miR-125b-5p, which are crucial inhibitors of osteoclastogenesis (Pascual-García et al., 2024). Numerous studies have found that miR-214 is overexpressed in the EVs of osteoclasts and plays a key role in regulating bone homeostasis by targeting the PI3K-AKT pathway (Yuan et al., 2018; Zhao et al., 2015).

Osteoclast precursors (Bone marrow macrophages stimulated with M-CSF and RANKL for 24 h) and osteoclasts (Bone marrow macrophages stimulated with M-CSF and RANKL for 120 h) both contribute to the hypertrophy of chondrocytes, with osteoclasts exhibiting a more significant promotional effect than osteoclast precursors. OC-EVs are considered an important pathway for osteoclast-chondrocyte crosstalk. OC-EVs can accelerate cartilage matrix degradation and promote chondrocyte hypertrophy through the inhibition of TGF-β1/SMAD2 signaling pathway by let-7a-5p and miR-212-3p (Dai et al., 2020; Dai et al., 2024). Studies have shown that in the early stages of OA, the miRNA expression profile of osteoclast-derived circulating exosomes undergoes changes. Specifically blocking the secretion of osteoclast exosomes can effectively alleviate the progression of OA. Researchers suggest that OC-EVs primarily accelerate articular cartilage damage by inhibiting TIMP-2 and TIMP-3 (Liu et al., 2021; Han, 2022).

### 2.3 Osteoblast-derived exosomes

During the process of bone formation, exosomes derived from osteoblasts (OB-Exos) play a positive-inductive role. OB-Exos can activate the osteogenic differentiation process and promote mineralization of osteosarcoma cells by targeting the URG4/WNT signaling pathway (Leng et al., 2024). The presence of key transcription factors involved in osteogenesis (RUNX2 and OSX) and miRNAs (miR-34a, miR-27a, and miR-22) that influence osteoblast differentiation within OB-Exos can enhance and accelerate the osteogenic differentiation of BMSCs (Narayanan et al., 2018). Exosomes derived from the mineralizing osteoblast cell line MC3T3-E1 are enriched with osteogenic miRNAs (miR-1192, miR-680, and miR-302a) and miRNAs that can target the Wnt/β-catenin pathway (miR-3084-3p, miR-680, miR-677-3p, and miR-5100), which can recruit and induce osteogenic differentiation of BMSCs (Cui Y. et al., 2016). Moreover, through miRNA-mRNA interactions, they participate in transcription initiation, matrix metalloproteinase inhibition, and cell cycle progression, thereby promoting the proliferation, migration, and differentiation of MC3T3-E1 cells (Samal et al., 2024). In addition, proteomic studies have revealed a significant upregulation of osteogenesis-related proteins such as TGFB3, BMP-1, SMURF-1, EIF2, TRIP-1, as well as annexins involved in calcium channels and WNT proteins in OB-Exos (Davies et al., 2019; Ge et al., 2017; Ge et al., 2015; de Oliveira et al., 2023). However, OB-Exos mediate a negative feedback mechanism at specific stages to prevent excessive bone formation. Studies have shown that mature osteoblast-secreted vesicles (primary osteoblasts expressing enhanced cyan fluorescent protein) are enriched with miR-143, which can target CBFB and inhibit RUNX2, thereby suppressing osteoblast differentiation and regulating the dynamic transition from the bone formation stage to the bone resorption stage (Uenaka et al., 2022). Nevertheless, to date, such research is still limited, and the mechanisms underlying the conversion between the osteoblast and osteoclast phases remain to be further elucidated.

OB-Exos participate in the bone resorption process through the RANKL-mediated signaling pathway, regulating the generation, differentiation, and functional activation of osteoclasts (Cappariello et al., 2018). In a dual-transgenic zebrafish fracture healing experiment, OB-Exos were phagocytosed by immature osteoclasts in response to fracture stress, thereby promoting their differentiation (Kobayashi-Sun et al., 2020). Transmission electron microscopy revealed the co-localization of TRAP and RANKL in osteoblast-derived vesicles (Solberg et al., 2015). Exosomes released from the osteoblast line UAMS-32P can induce nuclear translocation of NFATc1 through receptor-ligand interaction between the RANKL protein and RANK on RAW264.7 cells, thereby activating osteoclastic activity (Deng et al., 2015). OB-Exos release METTL14, which can enhance the m6A methylation level of NFATC1 to suppress osteoclast-induced bone resorption (Yang et al., 2023). Non-coding RNAs are also key factors in the regulation of bone resorption by OB-Exos. Osteoblasts pretreated with exosomes from mineralized osteoblasts can promote osteoclastogenesis, with the enriched miR-143-3p increasing the RANKL/OPG ratio in MC3T3-E1 cells (Uenaka et al., 2022). Circ_0008542 in MC3T3-E1 exosomes can sponge miR-185-5p, upregulating the expression of RANK. Additionally, the 1916–1992 bp fragment of circ_0008542 increases the level of m6A methylation, inhibits the RNA methyltransferase METTL3, and induces osteoclast differentiation and bone resorption (Wang W. et al., 2021). Concurrently, certain studies suggest that miR-125b released by OB-Exos inhibits osteoclast formation by targeting PRDM1 and suppresses the differentiation of RAW264.7 cells through the miR-503-3p/HPSE axis (Minamizaki et al., 2020; Wang Q. et al., 2021).

Subchondral bone is essential for maintaining cartilage homeostasis, and there is a significant correlation between the subchondral bone sclerosis induced by OB-Exos and cartilage degeneration (Maas et al., 2015; Li et al., 2021a; Larrouture et al., 2021; Zhou et al., 2019). Targeting specific signals in the subchondral bone, such as TGF-β, can alleviate the pathological severity of OA and reduce the pain response (Thielen et al., 2019; Cui Z. et al., 2016; Zhen et al., 2013). Wu et al. found that miR-210-5p is highly enriched in the exosomes derived from osteoblasts of OA sclerotic subchondral bone, triggering catabolic activity in chondrocytes and reducing the expression of chondrocyte-specific markers, playing a key role in the pathological progression of cartilage degeneration (Wu et al., 2021).

### 2.4 Osteocyte-derived exosomes

Osteocytes are mechanosensitive cells that can respond to external stimuli through paracrine actions, participating in the coordination of load-induced bone formation. Mechanical stimulation activates Ca^2+^ oscillations in osteocytes, enhancing the production and release of EVs containing bone regulatory proteins (Morrell et al., 2018), thereby promoting osteogenic differentiation and mineralization of the matrix (Nieuwoudt et al., 2021). Exosomes released by osteocytes (Ocy-Exos) exposed to mechanical strain significantly enhance the recruitment and osteogenic differentiation of human periodontal ligament stem cells and human bone marrow mesenchymal stem cells, which may be associated with the exosome-mediated miR-181b-5p/PTEN/AKT signaling pathway (Lv et al., 2020; Eichholz et al., 2020). Ocy-Exos can be rapidly absorbed by MC3T3 cells, and their enriched miR-218 can affect osteoblast activity by regulating SOST and WNT signaling pathways (Qin et al., 2017). Compared to EVs derived from senescent osteocytes (primary bone cells from 2-month-old mice), EVs derived from young osteocytes (primary bone cells from 16-month-old mice) significantly increase alkaline phosphatase activity, mineralization deposition, and the expression of osteogenesis-related genes in BMSCs, which may be due to the enrichment of TPM1 in EVs from young osteocytes, promoting osteogenesis (Wang Z. X. et al., 2024). Conversely, the reduced expression of miR-494-3p in exosomes derived from senescent osteocytes (senescent MLO-Y4 cells induced by tert-Butyl hydroperoxide) targets the PTEN/PI3K/AKT pathway, thereby inhibiting osteogenic differentiation (Yao et al., 2024).

As for the role of Ocy-Exos in bone resorption, no relevant studies have been retrieved to date. Kogianni and colleagues suggest that apoptotic bodies derived from osteocytes, independently of other osteoclastogenic factors, can specifically promote osteoclastogenesis and the initiation of bone resorption, while other soluble factors derived from osteocytes do not exhibit similar effects (Kogianni et al., 2008).

The communication mechanism between osteocytes and chondrocytes has not been fully elucidated. Only one study has shown that Ocy-Exos can activate the WNT pathway by upregulating DLX2 expression, alleviate IL-1β-induced cartilage damage, and enhance chondrocyte viability and migration ability (Xu et al., 2024). The precise communication mechanisms by which osteocytes transmit signals to other bone tissue cells are still poorly understood, and further exploration of these mechanisms is crucial for the development of innovative treatments for bone metabolic diseases.

### 2.5 Chondrocyte-derived exosomes

Chondrocytes can participate in bone remodeling through exosomal miR-221-3p. Studies have shown that miR-221-3p can be transferred to osteoblasts via exosomes, where it inhibits the expression of early osteogenesis markers COL1A1 and RUNX2 (Shang et al., 2021).

Chondrocyte-derived exosomes (CC-Exos) primarily target chondrocytes as their receptor cells, and they are released in a caspase-3 and Rho/Rock-dependent manner, closely associated with autophagy, playing a significant role in regulating cartilage metabolism (Rosenthal et al., 2015). Studies have indicated that CC-Exos can increase the expression of matrix synthesis markers ACAN, COL2A, and SOX9 in human umbilical cord mesenchymal stem cells, while decreasing the expression of chondrocyte hypertrophy and degeneration markers COL1A and COL10 (Ma et al., 2020). Low-intensity ultrasound stimulation can enhance the vesicular secretion of chondrocytes by altering the miRNA expression profile, and these vesicles can enhance the cellular activity, proliferation, migration, and chondrogenic differentiation of adipose-derived stem cells (Wang Y. et al., 2023). However, under OA pathological conditions, the properties of CC-Exos undergo a transformation. Dysfunctional CC-Exos promote M1 macrophage polarization and accelerate collagen degradation, which is related to their enrichment in lncRNA OANCT competitively binding to the FTO protein, thereby maintaining the mRNA stability of PIK3R5 and further activating the PI3K/AKT/mTOR pathway (Lv et al., 2022). Abnormal biomechanical loading alters the molecular levels of MGP, NPP1, and TNAP in CC-Exos, and increases exosome production, which leads to disorders of cartilage metabolism and ultimately results in cartilage degeneration and matrix calcification (Liu et al., 2022).

### 2.6 Macrophage-derived exosomes

Exosomes derived from bone marrow-derived macrophages (BMDM-exos) significantly increase the protein and mRNA levels of RUNX2, ALP, COLI, and OCN in BMSCs. Alizarin red and ALP staining also indicate an increased proportion of mineralized cells (Zhang D. et al., 2021). BMDM-exos can target the PPARα-ABCA1 axis through miR-378a, promoting the osteogenic differentiation of skeletal stem/progenitor cells (He et al., 2024). Macrophage polarization has a significant impact on the paracrine regulation of bone homeostasis (Kang et al., 2020). In most cases, exosomes derived from M2 macrophages (M2-Exos) act on various transcription factors, proteins, or hormones to promote osteogenesis, while exosomes derived from M1 macrophages (M1-Exos) inhibit osteogenesis (Bin-bin et al., 2022; Chen X. et al., 2022; Liao et al., 2022; Crippa et al., 2008). In M2-Exos, miR-690, miR-5106, and miR-378a are significantly overexpressed, and they can target IRS-1/TAZ, SIK2/SIK3, and BMP pathways or molecules, respectively, to promote osteogenic differentiation of BMSCs (Li et al., 2021b; Xiong et al., 2020; Kang et al., 2020). Additionally, they can inhibit TGF-β1 through targeting by miR-142-3p, which restores the normal osteogenic differentiation capacity of BMSCs after irradiation (Huang et al., 2024). Moreover, M2-Exos can also promote the osteogenic differentiation of LPS-induced periodontal ligament stem cells by enhancing the expression of CXCL12 (Gao and Wu, 2024). In contrast, M1-Exos can negatively regulate the BMP signaling pathway through miR-155 (Kang et al., 2020; Song et al., 2022). Additionally, studies have emphasized that alarm proteins carried by macrophage-derived vesicles may have a non-negligible impact on the function of osteoblasts and osteoclasts, warranting further investigation (Huang X. et al., 2022).

Compared to the extensive research on the role of macrophages in bone formation, there is a relative paucity of studies on macrophage involvement in bone resorption. Most studies suggest that M1-Exos exacerbate bone loss and osteoporosis. For instance, miR-155 contained in M1-Exos can inhibit the expression of BMP2, BMP9, and RUNX2; miR-98 can downregulate DUSP1 and activate the JNK signaling pathway; miR-222 can suppress the expression of the anti-apoptotic gene BCL-2; and miR-3470b can regulate the TAB3/NF-κB pathway, thereby promoting osteoclast differentiation and bone resorption *in vitro* (Kang et al., 2020; Yu L. et al., 2021; Qi et al., 2021; Pan et al., 2023). In contrast, M2-EVs significantly reduce the expression of osteoclastogenesis-related genes (such as CTSK, NFATC1, TRAP, miR-7, miR-1897) in RAW264.7 cells, thereby decreasing RANKL-induced osteoclast formation (Trentini et al., 2024; Zhou and Hu, 2024). Additionally, emerging evidence suggests that during the process of osteoclast-mediated bone resorption, osteomacrophages participate in the pathological process by absorbing byproducts, including bone particulate and TRAP (Batoon et al., 2021).

M2-EVs can transport macrophage reprogramming factors and proteins responsible for the generation and migration of M2 macrophages, thereby having a significant impact on articular cartilage metabolism (Kim et al., 2022). EVs derived from macrophages and osteoclasts can both induce the repolarization of macrophages towards the anti-inflammatory M2 phenotype, promoting chondrocyte function and cartilage formation (Li X. et al., 2024). Qian et al. found that exosomal miR-26b-5p from M2 macrophages can alleviate synovial inflammation and cartilage degeneration by targeting TLR3 and COL10A1, inhibiting chondrocyte hypertrophy (Qian et al., 2024). Cai and colleagues have discovered that M2-Exos may alleviate BMSC aging and enhance their chondrogenic potential by potentially regulating DNA replication and repair (Cai et al., 2024). Bai et al. indicated that the overexpression of lncRNA MM2P induces M2 macrophage polarization, which inhibits the dephosphorylation of STAT3 that is mediated by SHP2, and the resulting increase in p-STAT3 enhances the expression of SOX9 in M2-derived exosomes. This, through the delivery to chondrocytes via exosomes, significantly promotes matrix synthesis in chondrocytes (Bai et al., 2020).

In conclusion, the role of exosomes from cells within the skeletal microenvironment in bone homeostasis has been extensively investigated. Those derived from bone marrow mesenchymal stem cells and M2 macrophages facilitate the proliferation and differentiation of osteogenic lineage cells, promote the osteogenic differentiation of mesenchymal stem cells, and concurrently inhibit osteoclast generation and activation. Exosomes from osteoblasts and osteocytes primarily promote bone formation, while those from osteoclasts mainly facilitate bone resorption, collectively exerting a positive regulatory effect on bone growth and regeneration. Conversely, exosomes from M1 macrophages inhibit osteoblast differentiation and enhance osteoclast generation and activation. Exosomes from osteoclasts and chondrocytes primarily inhibit bone formation, whereas those from osteoblasts mainly promote bone resorption, thus collectively playing a negative regulatory role in bone remodeling to maintain the dynamic equilibrium of bone tissue. Regarding cartilage metabolism, exosomes from bone marrow mesenchymal stem cells, osteocytes, M2 macrophages, and chondrocytes contribute to chondrocyte proliferation and migration, enhancing chondrocyte viability, inhibiting cellular degeneration and apoptosis, and improving cartilage metabolism. However, exosomes from osteoblasts and osteoclasts may promote chondrocyte hypertrophy and degeneration, accelerating cartilage matrix degradation.

## 3 Exosome derived from cells outside the bone microenvironment

Recently, exosomes derived from cells outside the skeletal microenvironment have garnered extensive attention, with studies emphasizing their ease of acquisition and potent bone-targeting capabilities (Wang P. et al., 2024). This section systematically reviews the regulatory mechanisms of exosomes from cells outside the skeletal microenvironment in maintaining bone homeostasis (illustrated in Figures 1–3), primarily involving stem cells derived from non-osseous tissues (including umbilical cord mesenchymal stem cells, adipose-derived stem cells, endothelial progenitor cells, and synovial mesenchymal stem cells), vascular-derived cells (including endothelial cells and pericytes), muscle-derived cells (myoblasts), as well as neurogenic cells (Schwann cells).

### 3.1 Exosomes derived from stem cells other than BMSCs

#### 3.1.1 Exosomes derived from umbilical cord mesenchymal stem cells

Exosomes derived from umbilical cord mesenchymal stem cells (UCMSC-EVs) can enhance the regenerative capacity of recipient cells, BMSCs, in bone formation through the transfer of cell cycle-related protein PCNA and osteogenic protein CLEC11A (Lei et al., 2021; Hu Y. et al., 2020). In a glucocorticoid-induced avascular necrosis of the femoral head disease model, UCMSC-Exos can reduce the apoptosis level of MC3T3-E1 cells by inhibiting the MAPK signaling pathway and ROS levels, or by activating the Hippo signaling pathway through miR-365a-5p, thereby promoting a significant upregulation of osteogenic genes such as BMP2, SP7, and RUNX2 in the target bone tissue (Lu et al., 2023; Kuang et al., 2020).

UCMSC-EVs exert an anti-resorptive effect through CLEC11A mediation, significantly reducing the levels of osteoclast differentiation markers such as NFATC1, TRAP, and CTSK in RAW264.7 cells, and inhibiting the formation of mature osteoclasts (Hu H. et al., 2020). Additionally, intervention with UCMSC-EVs can decrease the levels of ROS and inflammatory cytokines in RAW264.7 cells, suppressing PE + RANKL-induced osteoclast differentiation (Xie et al., 2023).

UCMSC-EVs have been demonstrated to exert anti-inflammatory effects in OA models. They can reverse the abnormal expression of COL2A1 and MMP13 induced by IL-1β in chondrocytes, protect articular cartilage, and inhibit secondary cartilage degeneration. This protective and regenerative effect is mediated by key miRNAs such as miR-23a-3p and miR-1208, which target signaling pathways including p53, AKT, and NLRP3, thereby promoting cartilage regeneration. The ability of UCMSC-EVs to modulate these pathways suggests their potential as a therapeutic intervention for OA by reducing inflammation and promoting the repair and regeneration of damaged cartilage (Pan et al., 2024; Wang S. et al., 2023; Cao et al., 2023; Hu Y. et al., 2020; Zhou et al., 2022).

#### 3.1.2 Exosomes derived from adipose-derived stem cells

Exosomes derived from adipose-derived stem cells (ADSC-Exos) have also been extensively studied for their application in skeletal regeneration and repair (Chen J. et al., 2022). Studies have shown that ADSCs-Exos can alleviate TNF-α-induced cytotoxicity and apoptosis in primary osteoblasts (Wang S. Z. et al., 2021). Furthermore, Li et al. suggest that compared to exosomes derived from bone marrow stem cells and synovial stem cells, ADSC-EVs exhibit superior capabilities in promoting the migration, proliferation, and osteogenic differentiation of BMSCs, as well as inducing bone regeneration in mouse models (Li Q. et al., 2021). The potential mechanisms by which ADSC-EVs regulate bone formation may include focal adhesion, ECM-receptor interaction, actin cytoskeleton, cAMP, PI3K-AKT, and Wnt/β-catenin signaling pathways (Li et al., 2021a; Zhang D. et al., 2023). The regulatory mechanism of miRNAs is also not to be overlooked. Li et al. found that ADSC-Exos promote the polarization of M1 macrophages to the M2 phenotype through the inhibition of MIF by miR-451a, thereby enhancing downstream osteoblast function (Li R. et al., 2022). Additionally, exosomal miR-130a-3p, which is significantly upregulated during the osteogenic differentiation of ADSCs, can promote this process by mediating the WNT signaling pathway (Yang S. et al., 2020).

ADSC-Exos play a pivotal role in modulating osteoclast activity through a variety of mechanisms. Ren et al. found that ADSC-Exos effectively alleviate hypoxia- and serum deprivation-induced osteocyte apoptosis, while also reducing the ratio of RANKL to OPG, thereby inhibiting osteocyte-mediated osteoclastogenesis (Ren et al., 2019). ADSC-Exos also inhibit the activation of the NLRP3 inflammasome within osteoclasts, diminishing bone resorption and contributing to the recovery from bone loss (Zhang L. et al., 2021). Within ADSC-Exos, OPG, miR-21-5p, and let-7b-5p are abundant, and they significantly suppress osteoclast differentiation and reduce the expression of genes associated with bone resorption through the inhibition of RANKL and ACVR2A (Lee et al., 2021).

ADSC-Exos inhibit the degradation of articular cartilage by promoting chondroanabolic metabolism and preventing chondrolytic metabolism. Studies have demonstrated that ADSC-Exos reduce the expression of MMP-13, caspase-1, and IL-1 in IL-1β-induced chondrocytes and significantly improve the condition of articular cartilage in animal models. They can decrease the loss of proteoglycans and promote matrix synthesis, as well as increase the expression of collagen type II and aggrecan both *in vitro* and *in vivo* (Zhao et al., 2023; Xu et al., 2022; Liu Q. et al., 2024). MiRNAs serve as a crucial pathway for the functional effects of ADSC-Exos. Evidence has shown that ADSC-Exos, through miR-376c-3p, target Wnt3 or Wnt9a to inhibit the Wnt/β-catenin pathway, thereby alleviating chondrocytes degradation caused by OA (Li F. et al., 2023). Additionally, research indicates that ADSC-Exos can target and inhibit RUNX2 through miR-338-3p, effectively repairing the inflammatory damage induced by IL-1β in the murine chondrogenic cell line ATDC5 and promoting cell proliferation (Li C. et al., 2022). However, in contrast to normal ADSCs, aged ADSCs (etoposide-induced senescent cells) induce a catabolic and pro-inflammatory gene signature in mouse joints and fail to mitigate collagenase-induced chondrocyte damage (Boulestreau et al., 2024).

Some studies have demonstrated that osteoarthritis ().

#### 3.1.3 Exosomes derived from endothelial progenitor cells

Exosomes derived from endothelial progenitor cells (EPC-Exos) do not directly promote the osteogenic differentiation of BMSCs (Qin and Zhang, 2017). Therefore, some studies suggest that EPC-EVs may primarily facilitate bone regeneration indirectly by accelerating angiogenesis (Bouland et al., 2021). In the research by Lu and Chen, it was confirmed that EPC-Exos can exert osteogenic effects through the osteoblast lineage (Lu et al., 2019; Chen G. H. et al., 2019). Studies have shown that EPC-Exos can inhibit glucocorticoid-induced oxidative damage and ferroptosis in osteoblasts through the miR-126-mediated ERK1/2/BCL-2 pathway, thereby promoting bone regeneration.

EPC-Exos also participate in promoting bone repair by enhancing the recruitment and differentiation of RAW264.7 cells. The mechanism of action may be related to the interaction between lncRNA-MALAT1 and miR-124 within EPC-Exos (Cui et al., 2019; Ohnuma et al., 2019).

To date, no studies have explored the role of EPC-Exos in cartilage metabolism; however, given their potent anti-inflammatory and reparative properties (Yuan et al., 2023), this area of research holds significant potential.

#### 3.1.4 Exosomes derived from synovial mesenchymal stem cells

Exosomes derived from synovial mesenchymal stem cells (SMSC-Exos) primarily influence cartilage metabolism. They can promote the migration and proliferation of chondrocytes, improve matrix metabolism, reduce IL-1β-induced cellular inflammation and apoptosis, and facilitate chondrogenesis *in vitro*, thereby reducing the destructive effects of OA on articular cartilage. This may be associated with the miR-320c-mediated ADAM19/WNT pathway, miR-130b-3p-mediated LRP12/AKT/β-catenin pathway, miR-26a-5p/PTEN axis, MATN3/IL-17A-mediated PI3K/AKT/mTOR pathway, and the CXCL5/CXCL6 axis (Zhu et al., 2017; Kong et al., 2022; Kong et al., 2023; Zeng et al., 2022; Lu et al., 2021; Long et al., 2023; Kawata et al., 2021). Overexpressing the chondroprotective miRNAs in SMSC-Exos can further enhance their protective effects. Tao et al. have revealed that SMSC-Exos overexpressing miR-140-5p can promote cartilage regeneration and delay the progression of knee OA (Tao et al., 2017). Wang et al. found that SMSC-Exos overexpressing miR-155-5p promote chondrocyte migration and proliferation, inhibit apoptosis, enhance extracellular matrix secretion, and effectively prevent cold-induced OA (Wang Z. et al., 2021). Wang et al. indicated that SMSC-EVs overexpressing miR-31 enhance chondrocyte proliferation and migration by targeting KDM2A, alleviating cartilage damage and inflammation in the knee (Wang et al., 2020). Additionally, LPS appears to further enhance the therapeutic effects of SMSC-Exos on OA, which may be related to the high expression of miRNAs induced by LPS (Duan et al., 2021).

### 3.2 Exosomes derived from vascular cells

Studies have indicated that exosomes derived from vascular endothelial cells (EC-Exos) possess potent bone-targeting properties. They can reverse steroid-induced osteogenic inhibition by inhibiting ferroptosis, which is dependent on ferritin autophagy (Yang et al., 2021a). EC-Exos can deliver NEAT1 through the DDX3X/NLRP3 axis to promote bone generation mediated by M2 polarized macrophages (Chen et al., 2023). Additionally, they can inhibit osteoclast activity by upregulating miR-155 (Song et al., 2019). However, the impact of EC-Exos on cartilage metabolism is detrimental; they reduce the ability of chondrocytes to resist oxidative stress by inhibiting autophagy and P21 expression, thereby increasing cellular ROS levels and inducing apoptosis (Yang et al., 2021b).

Exosomes derived from vascular pericytes negatively regulate osteoclast development and bone resorption, primarily through the inhibition of the TRAF3-mediated NF-κB pathway (Cai et al., 2023).

Exosomes derived from vascular smooth muscle cells are enriched with protein transcripts linked to the osteocyte phenotype, as well as molecules involved in the WNT/β-catenin signaling pathway. These exosomes can promote the differentiation of BMSCs into osteocytes, thereby contributing to improved bone regeneration (Fernandes et al., 2024).

### 3.3 The exosomes of muscle-derived cells

Exosomes of myogenic origin play a role in promoting bone formation. Studies have shown that myoblasts C2C12 can increase the level of miR-27a-3p in MC3T3-E1 cells, thereby reducing APC expression, activating the β-catenin pathway, and promoting the differentiation of MC3T3-E1 cells into osteoblasts (Xu Q. et al., 2018). Additionally, they can transfer PRRX2, which promotes the transcriptional activation of MIR22HG by sequestering miR-128, thereby activating the YAP pathway and enhancing the osteogenic differentiation of BMSCs (Li Y. et al., 2023). Mechanical stress increases the secretion of exosomes from C2C12 cells and enhances their osteogenic effect. Xu et al. suggest that exosomes derived from C2C12 cells induced by mechanical stress can promote the proliferation and osteogenic differentiation of BMSCs through the miR-92a-3p/PTEN/AKT signaling pathway (Xu et al., 2023). However, after inducing senescence in C2C12 cells using hydroxides, the extracted exosomes reduced the activity of BMSCs and increased the senescence of BMSCs, which may be associated with the elevated levels of miR-34a in the exosomes (Fulzele et al., 2019).

### 3.4 Exosomes derived from neurogenic cell

Schwann cells, a type of specialized glial cell primarily found in the peripheral nervous system, have recently garnered significant attention for their regulatory role in bone formation. Numerous studies have demonstrated that Schwann cells can promote the migration, proliferation, and differentiation of BMSCs through exosome-mediated communication, with the underlying mechanisms primarily involving the let-7c-5p/TGF-β pathway and the TGF-β1/SMAD2/3 pathway (Wu et al., 2020; Wang T. et al., 2023; Hao et al., 2022). Compared to exosomes from other sources, there is a paucity of research on the regulation of bone homeostasis by neurogenic exosomes. However, in recent years, a growing number of researchers have focused on the neuro-skeletal axis, emphasizing the interactions between the nervous and skeletal systems, as well as the contributions of neural influences on skeletal metabolism, homeostasis, and injury repair. It is believed that research related to neurogenic exosomes will be further expanded in the near future (Li J. et al., 2024; Wan et al., 2021; Chen et al., 2024).

In short, exosomes derived from cells outside the skeletal microenvironment exert a targeted regulatory effect on bone tissue through distant actions, influencing bone metabolism. Those derived from umbilical cord mesenchymal stem cells, adipose-derived stem cells, and vascular endothelial cells both stimulate bone formation and suppress bone resorption. Exosomes from endothelial progenitor cells, vascular smooth muscle cells, and myoblasts mainly enhance bone formation, while those from vascular pericytes primarily inhibit bone resorption, collectively facilitating bone growth and regeneration. Additionally, endothelial progenitor cell-derived exosomes also contribute to bone repair by boosting the recruitment and differentiation of osteoclast precursors. Regarding cartilage metabolism, exosomes from umbilical cord mesenchymal stem cells, adipose-derived stem cells, and synovial mesenchymal stem cells promote cartilage matrix synthesis and prevent degradation, whereas vascular endothelial cell-derived exosomes negatively impact cartilage metabolism by accelerating chondrocyte apoptosis.

## 4 Conclusion

In summary, exosomal communication plays a pivotal role in maintaining bone homeostasis by facilitating the interplay between various cells within the bone microenvironment and outside through the delivery of bioactive molecules. This process regulates bone formation, bone resorption, and cartilage metabolism. In-depth investigation of the mechanisms underlying exosomal communication is instrumental in unraveling the pathogenesis and development of bone metabolic diseases, thereby contributing to the development of more scientific, effective, and innovative therapeutic strategies.

It is noteworthy that there exist opposing conclusions in the research on the efficacy of exosomes derived from osteoblasts and osteoclasts in bone formation and bone resorption. In response to this phenomenon, we believe that it is essential to first eliminate the interference caused by exosomal heterogeneity resulting from different isolation techniques and preprocessing methods. Certainly, OB-Exos and OC-Exos are expected to have negative feedback mechanisms to prevent excessive bone formation or resorption, regulating the dynamic transition between the bone formation and bone resorption phases. This is obviously related to the maturity stage of osteoblasts and osteoclasts. However, to date, due to the complexity of exosomal components and their multi-target effects, the regulatory mechanisms of the exosomal network in bone turnover remain to be elucidated.

Furthermore, external factors such as mechanical stimulation, aging, and inflammation have a profound impact on the release and biological effects of exosomes. However, how these complex factors precisely regulate the secretion of exosomes, the selection of their contents, and their functions in target cells remains a challenging area of scientific research.

Compared to the well-studied exosomes derived from cells within the skeletal microenvironment, research on exosomes from extra-osseous cells remains limited. Through this review, We have highlighted that bone tissue and extra-osseous tissue, using exosomes as a medium, form a mutually influential entity that collectively plays a significant role in the regulation of bone homeostasis. We also use this opportunity to call for greater attention to the distant communication effects of exosomes in future research.

Exosomes, as key regulators of bone homeostasis, are also involved in the pathological processes of bone metabolic diseases such as OP and O Numerous studies have confirmed that active components of traditional Chinese medicine can regulate bone and cartilage metabolism by affecting the production, release, or composition of exosome contents (Wu et al., 2022; Huang M. Z. et al., 2022; Yan et al., 2024). Exosomes serve as an important target for their therapeutic effects in bone metabolic diseases. This understanding provides new insights and inspiration for more effectively utilizing exosomes to modulate bone homeostasis and develop novel treatment methods in the future.

Exosomes exhibit favorable biocompatibility, stability, targeting efficiency, as well as low immunogenicity and toxicity (Wang C. et al., 2023). Owing to their lipid bilayer structure, they can readily penetrate biological barriers and are considered a significant cell-free therapeutic approach in bone metabolic disorders. As vectors for drug delivery, the combination of exosomes with biomaterials or the modification of exosomal contents through genetic engineering, biochemical methods, or other technical strategies represent the three major research directions in the field of exosome-based therapeutics (Vig and Fernandes, 2022). Preclinical models have already confirmed their substantial clinical potential. Although exosome therapy holds great promise in clinical applications, there is still a long way to go before it can be practically applied in clinical practice. Currently, there are no clinical trials of exosome therapy for the treatment of osteoporosis or osteoarthritis in the ClinicalTrials database (https://clinicaltrials.gov/), and no relevant research papers can be found in the PubMed database. Moving forward, it is imperative to continue advancing clinical trials to comprehensively evaluate the true clinical value of exosomes in the management of bone metabolic diseases.

As mentioned above, despite the recognized key role of exosomes in bone metabolism, the underlying molecular mechanisms and signaling pathways are still poorly understood. Additionally, technical challenges in the field of exosome research, such as efficient isolation and purification, precise identification, and compositional analysis, are also obstacles that need to be addressed. In the future, efforts must be made to achieve technological innovation and method standardization. Through multidisciplinary collaborations involving molecular biology, bioinformatics, nanomedicine, materials science, and other fields, we aim to further elucidate the network regulatory mechanisms of exosomes in bone homeostasis. This will enable us to better understand and harness the potential value of exosomes in disease diagnosis, treatment, and prevention.
